# The regulatory mechanism of p38/MAPK in the chondrogenic differentiation from bone marrow mesenchymal stem cells

**DOI:** 10.1186/s13018-019-1505-2

**Published:** 2019-12-12

**Authors:** Ning Ma, Xiao Teng, Qi Zheng, Peng Chen

**Affiliations:** 1 Department of Orthopedics, Zhejiang Taizhou Central Hospital (Affiliated Hospital of Taizhou University), No. 999 Donghai Avenue, Jiaojiang District, Taizhou, 318000 Zhejiang China; 2 Department of Orthopedics, Yan Cheng Third People’s Hospital (Affiliated Yancheng Hospital of Southeast University Medical College), No.2 Xindu West Road, Yancheng, 224001 Jiangsu China

**Keywords:** Osteoarthritis, Chondrogenic differentiation, p38-MAPK, BMSCs, TGF-β1

## Abstract

**Background:**

Osteoarthritis (OA) is a degenerative joint disease characterized by articular cartilage degradation and joint inflammation, in which growth factors are significantly involved. The extracellular signal-regulated p38 MAPK pathways play important roles in the regulation of osteogenic and chondrogenic differentiation in bone marrow mesenchymal stem cells (BMSCs). However, the exact mechanism remains unclear.

**Methods:**

In this study, the chondrogenic differentiation of human BMSCs was initiated in micromass culture in the presence of TGF-β1 for 14 days. Quantitative RT-PCR and Western blot were performed to detect the transfection effect of shRNA-p38 interfering plasmid in BMSCs. The protein expressions of p/t-p38, SOX9, collagen II, Aggrecan, p/t-Smad1, and p/t-Smad4, as well as the kinase activities of p38/ERK/JNK pathway, were investigated using Western blot analysis. Additionally, the level of chondroitin sulfate and glycosaminoglycans (GAG) expression were measured by Alcian blue staining and GAG assay kit via qualitative and quantitative methods, respectively.

**Results:**

The results demonstrated that p38 pathway was activated in the chondrogenic differentiation of BMSCs induced by TGF-β1. Cartilage-specific genes and chondrogenic regulators, such as SOX9, collagen II, Aggrecan, and GAG, were upregulated by TGF-β1, which could be reversed by predisposed with shRNA-p38 interfering plasmid and p38-MAPK inhibitors (SB203580). Moreover, the activation of p38/ERK/JNK pathways in the presence of TGF-β1 was suppressed by shRNA-p38 and SB203580 treatment.

**Conclusion:**

Collectively, the activation of p38/ERK/JNK/Smad pathways plays a facilitated role in the chondrogenic differentiation induced by TGF-β1. After suppressing the p38 pathway, the chondrogenesis can be inhibited, which can be used to guide the treatment of osteoarthritis.

## Introduction

Osteoarthritis (OA) is a chronic joint disorder, which has been the leading cause of disability worldwide, particularly in the elderly suffering from pain and locomotor limitation [1–3]. It was proved to primarily occur in the hip and knee and results from the increase of age, obesity, strain, and injury [4, 5]. Chondrogenesis is mainly inducted by mesenchymal stem cell (MSC) condensation and differentiation into chondrocytes, which is regarded as the clinical treatment of OA [6, 7]. In addition, bone marrow MSCs possess the ability of multi-differentiation, such as chondrocytes, osteoblasts, and adipocytes, and have been the common seed cells for clinical research and therapy in orthopedics field [8]. However, the chondrogenic differentiation was hardly spontaneous; thus, it is necessary for the researchers to reveal the mechanisms underlying the differentiation from MSCs to chondrocytes, which is an important contribution for cartilage injury repair in OA.

Chondrogenic induction is conducted by forcing aggregation of mesenchymal cells or chondroprogenitor cells to generate a “micromass” or “pellet” culture in a special culture system in vitro [9–11]. TGF-β has been widely proven to regulate cell differentiation by setting off a large variety of signaling cascades and is tightly controlled by feedback mechanisms at different levels [12]. Mitogen-activated protein kinase (MAPK) family transduction involves a multistep kinase cascade with extracellular signal-regulated protein kinase (ERK), p38 kinase, and c-Jun N-terminal kinase (JNK) [13]. ERK and p38 have been confirmed to occupy core positions in mediating chondrocyte proliferation and related gene expression [14]. Further, MAPKs have been reported as the most important signaling pathway protein kinases, implicated in TGF-β-mediated chondrocyte functions including cell proliferation, differentiation, apoptosis, and inflammatory responses [15]. However, the definite effects and the potential mechanism of ERK and p38 in chondrogenesis are still unclear.

TGF-β triggers a series of downstream pathways via a transmembrane serine/threonine kinase receptor complex, known to be quite complicated. Smads are regarded as central cytoplasmic mediators of TGF-β [16, 17]. Upon TGF-β activation, Smad2 and Smad3 are activated to form protein complexes with common mediator Smad4, and then phosphorylated and translocated into nucleus to regulate transcription of target genes such as Sox9, Col2, and Aggrecan [18]. Recently, it was reported that p38 and Smad1/5/8 can be simultaneously activated by TGF-β-activated kinase 1 (TAK1), resulting in the induction of chondrogenic differentiation of stem cells [19]. These findings implied that chondrogenesis initiated by TGF-β1 may be tightly regulated through interactions between Smads and p38 MAPK signals. However, the mechanism of p38 pathways in TGF-β-induced chondrogenesis has not been fully clarified.

In the present study, we employed a micromass chondrogenic culture system for a relatively long duration of 14 days. We sought to investigate the function of p38/MEK/ERK/JNK signal pathway in TGF-β-induced chondrogenesis via regulating cartilage-specific genes for collagen II, Aggrecan, Sox9, and sGAG, and cross-talk between p38 MAPK and Smad1/4 signals in this process.

## Materials and methods

### Cell culture and reagent

Human bone marrow mesenchymal stem cells (BMSCs) were purchased from the American Type Culture Collection and cultured in α-modified Eagle medium (Invitrogen, Carlsbad, CA, USA) containing 15% FBS (Invitrogen), 100 mM l-ascorbic acid 2-phosphate (Wako Pure Chemical Industries, Osaka, Japan), 1% penicillin and streptomycin (Sigma), and 1% l-glutamine (Invitrogen). The cells were incubated at 37 °C in a humidified incubator with 5% CO_2_. When hBMSCs were 80–90% confluent in the culture flasks, the adherent cells were digested with trypsin and subcultured.

### Chondrogenesis induction in vitro

Chondrogenic differentiation of BMSCs was initiated in a micromass culture system. hBMSCs (at passages 3–6) were dissociated for single-cell suspension stating at a density of 2.0 × 10^7^ cells/ml. Ten-microliter droplets were seeded in culture dishes and allowed to form cell aggregates at 37 °C. In the experimental groups, recombinant human TGF-β1 (10 ng/ml) was added to the chondrogenic medium. Further, micromass cultures were performed for 14 days, and culture media was replaced every 3 days.

### Cell transfection and treatment

Transient transfections of primary chondrocytes were performed as described using Fugene (Roche) [20]. BMSCs were transfected when 60% of them were fused. The cells were transfected with a shRNA-p38 or an empty expression vector as shRNA-negative control (NC) (Invitrogen), or predisposed with the pharmacological p38 MAPK inhibitors (SB203580, 10 μM) after TGF-β1 inducing for 14 days. All the transfections were performed with three independent cell isolations, each of which has quadruplicate replicates.

### Real-time quantitative PCR

Total RNA was isolated from MPC pellets using the RNeasy Mini Kit (Qiagen, Valencia, CA) and quantified spectrophotometrically based on A260. After the first-strand cDNA synthesis using the SuperScript First-Strand Synthesis System (Invitrogen), levels of specific mRNA transcripts were determined by real-time polymerase chain reaction (PCR) utilizing SYBR Green PCR Master Mix and an iCycler real-time PCR detection system (Bio-Rad Laboratories). The primer sequences were as follows: p-p38 forward: 5′-CTGAACAACATCGTCAAGTGCC-3′, reverse: 5′-CATAGCCGGTCATCTCCTCG-3′; p38 forward: 5′-, CCCGAGCGTTACCAGAACC-3′, reverse: 5′- TCGCATGAATGATGGACTGAAAT-3′; GAPDH, forward: 5′-GCACCGTCAAGGCTGAGAAC-3′, reverse: 5′-TGGTGAAGACGCCAGTGGA-3′. The 2^−ΔΔCt^ method was used in each sample as relative quantification [21]. Each experiment was performed three times.

### Western blot analysis

The BMSCs were collected and lysed at 48 h after treatment using RIPA buffer. Identical amounts of proteins were resolved by 10–15% SDS-PAGE and then transferred to a PVDF membrane (Millipore Corporation, Billerica, MA, USA). The membrane was incubated with specific antibodies, Collagen II, Aggrecan, Sox9, p-p38, p38, p-ERK, ERK, p-JNK, JNK, p-Smad1, Smad1, p-Smad4, Smad4, and GAPDH (all from Cell Signaling Technology Inc.) at 4 °C overnight, and peroxidase-conjugated secondary antibodies (KPL, Gaithersburg, MD, USA). Chemiluminescence (Millipore Corporation) and densitometry analysis (ImageJ software) were applied to measure the protein expression. Each experiment was performed three times.

### Alcian blue staining

To assess the presence of glycosaminoglycans, which are cartilage-specific matrix proteins, BMSCs were cultured for 14 days in chondrogenic medium and stained with Alcian blue. After removal of the culture medium, the cell-seeded hydrogels were fixed with 4% paraformalydehyde for 30 min, then washed twice with PBS before the addition of 0.1% stock solutions of Alcian blue. After 30 min incubation at room temperature, the dye solution was removed and the constructs were washed with distilled water. Moreover, the staining results were recorded under an inverted microscope (Nikon Eclipse TS100). Each experiment was performed three times.

### Sulfated glycosaminoglycan quantification

Colorimetric assessment was performed to determine the amount of the sulfated glycosaminoglycan (sGAG) using a blyscan sulfated glycosaminoglycan assay kit (Biocolor B1000 Std.) according to the manufacturer’s instructions. Briefly, 0.25 ml of 1,9-dimethyl-methylene blue (DMMB) dye reagent was added to 80 μl of papain-digested extracts and incubated for 30 min at room temperature. The unbonded dye was removed by centrifugation. Then, the sGAG-bonded dye pellet was dissolved in 0.25 ml of dissociation reagent buffer. A serial dilution of chondroitin 4-sulfate (200 to 1500 ng) was prepared to generate a standard curve according to the same procedure used for sample preparation. The absorbance was recoded at 525 nm using a microplate reader (Multiskan Spectrum, Thermo Scientific). Each experiment was performed three times.

### Statistical analysis

Statistical analyses were carried out using Prism 5 software. Each experiment was performed in triplicate and analyzed by one-way ANOVA, followed by Dunnett’s post hoc test. Student’s *t* test was used to compare the values of the test and control samples. The results were expressed as mean ± SD. In all cases, a *p* value of < 0.05 was considered to be statistically significant.

## Results

### Activation of p38 in BMSCs after TGF-β1 stimulation

**Fig. 1:**
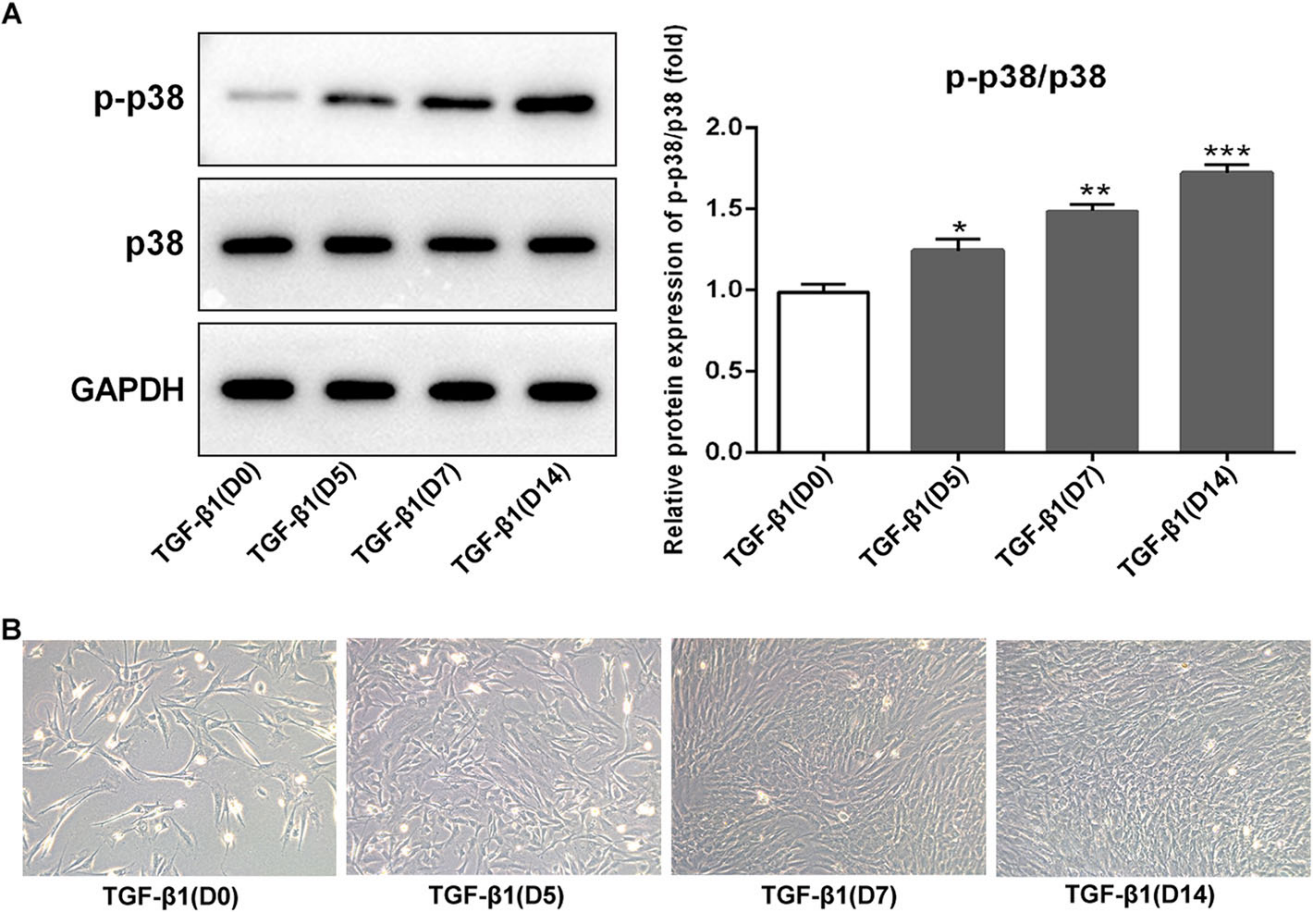
Expression of p-p38/p38 in TGF-β1-induced BMSCs and morphological observation. **a** Western blotting of p-p38 and p38 expression in BMSCs following TGF-β1-induced for 0, 5, 7, and 14 days. Relative protein expression of p-p38/p38 was elevated on a time-dependent manner under the TGF-β1 induction when compared with day 0. **b** Representative images of BMSCs. Morphological changes of BMSCs following TGF-β1 induced for 0, 5, 7, and 14 days were observed under an inverted microscope. On day 0, BMSCs grew adherently to the wall, and the cells were triangular or polygonal in shape. From day 5 to day 14, cell morphology changed significantly and gradually presented a typical paving stone shape with uniform size and shape. *p < 0.05, **p < 0.01, and ***p < 0.001 vs. TGF-β1 (D0)

To investigate whether p38 signal was involved in chondrogenesis induced by TGF-β1, the expression of p38 was examined by Western blot analysis. Results showed that the expression of phosphorylated (p)-p38 was exhibited at a low level on day 0 after adding TGF-β1 (10 ng/ml), but gradually increased as chondrogenesis proceeded, and reached at a relatively high level until day 14 compared to day 0 (Fig. 1a, *p* < 0.05 at day 5, *p* < 0.01 at day 7, and *p* < 0.001 at day 14). The p-p38 expression in TGF-β-induced BMSCs was significantly increased in a time-dependent manner. However, the expression of p38 showed no significant difference during 14 days. Therefore, these results indicated that the p38 signal was activated in chondrogenic differentiation of the BMSCs induced by TGF-β1.

As shown in Fig. 1b, at day 0 after adding TGF-β1, BMSCs grew adherently to the wall, and the cells were triangular or polygonal in shape. From day 5 to day 14, cell morphology changed significantly and gradually presented a typical paving stone shape with uniform size and shape.

### Inhibition of p38 signals suppressed the chondrogenic differentiation in TGF-β1-induced BMSCs

**Fig. 2:**
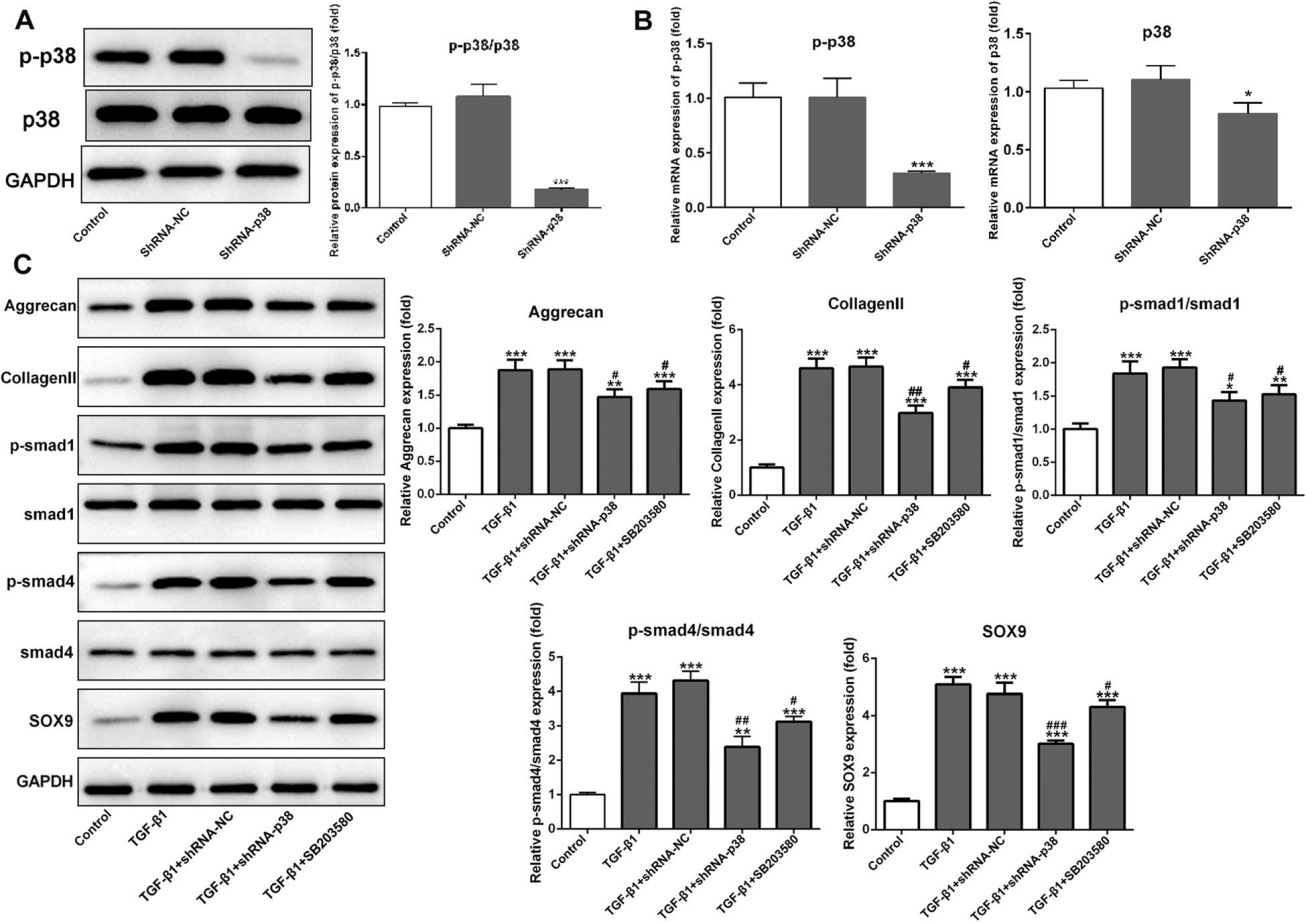
Downregulation of p38 inhibited the expression of chondrocyte-specific genes, such as p-smad1/smad1 and p-smad4/smad4 in TGF-β1-induced BMSCs. **a** Western blotting indicated that the protein expression of p-p38 was sharply decreased in BMSCs after transfecting with shRNA-p38 with no change on the p38 protein level. **b** The mRNA level of p-p38 and p38 was decreased in the shRNA-p38 group when compared with control and shRNA-NC groups from the results of qRT-PCR assays. **c** Representative pictures and statistical analysis results of Western blot showed that the expression of Aggrecan, collagen II, p-smad1/smad1, p-smad4/smad4, and SOX9 notably reduced in the TGF-β1-induced BMSCs when compared with control, which were upregulated by shRNA-p38 and SB203580 significantly. *p < 0.05, **p < 0.01, and ***p < 0.001 vs. control; ^#^*p* < 0.05, ^##^*p* < 0.01, and ^###^*p* < 0.001 vs. TGF-β1

The overexpression of p-p38 in TGF-β-induced BMSCs indicated that p38 signal pathway might function as an enhancer of the chondrogenic differentiation. To test this hypothesis, we investigated whether inhibition of p38 affects chondrogenic differentiation in TGF-β-induced BMSCs. In Fig. 2 a and b, the results showed that the protein level of p-p38 and the mRNA of p38 were significantly decreased using Western blot and RT-qPCR after being transfected with p38 interfering plasmid (shRNA-p38) in TGF-β-induced BMSCs (*p* < 0.001). Interestingly, the protein expression of p38 was almost unchanged (Fig. 2a), while its mRNA level decreased significantly in the shRNA-p38 group when compared with control and shRNA-NC groups (Fig. 2b, *p* < 0.05). Chondrogenic potential of BMSCs was confirmed by examining the expression of cartilage-specific gene coding for collagen II, Aggrecan, Sox9, p/t-smad1, and p/t-smad4 proteins with Western blot. TGF-β-treated BMSCs exhibited higher expression in all these cartilage-specific genes after 14 days compared with the untreated control group (Fig. 2c, *p* < 0.001). Treatment of shRNA-p38 and pretreatment of SB203580, TGF-β1-induced gene expression of collagen II, Aggrecan, Sox9, p/t-smad1, and p/t-smad4 were notably decreased compared with the TGF-β1 group (Fig. 2c). Further, the reverse effects of transfection interference plasmids shRNA-p38 were better than that of p38 inhibitors SB203580. Thus, these results indicated that cartilage-specific gene expression was affected by p38 inhibitors in reverse patterns, confirming that p38 could regulate the process of chondrogenic differentiation.

**Fig. 3:**
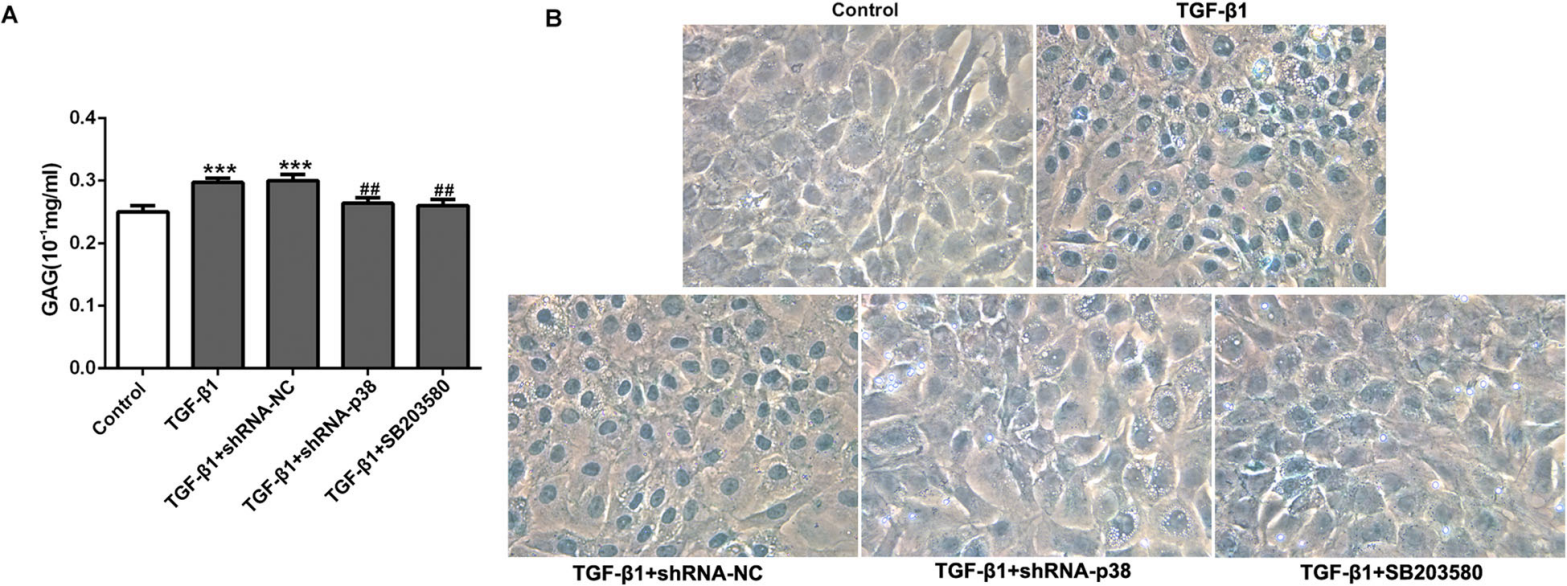
Effects of p38 inhibition on GAG level and the degree of chondrocyte differentiation in TGF-β1-induced BMSCs. **a** GAG assay and **b** Alcian blue staining showed that the GAG level and the number of blue-stained cells (the nuclei of chondrocytes can be blue-stained) in BMSCs were distinctly increased in TGF-β1-induced BMSCs, while p38 silence inhibited chondrogenic differentiation significantly. ***p < 0.001 vs. control; ^##^*p* < 0.01 vs. TGF-β1

In addition, a quantitative method was utilized to measure the amount of sulfated glycosaminoglycan (sGAG) deposited in the extracellular matrix (ECM) of the micromasses. The results showed a significant increase with TGF-β1 group in sGAG content compared to the control group (Fig. 3a, *p* < 0.001), while TGF-β1 in combination with shRNA-p38 or SB203580 exhibited a significant decrease in sGAG content compared to the TGF-β1 group at day 14 (Fig. 3a, *p* < 0.01). Therefore, GAG content was significantly regulated by the p38 pathway in TGF-β1-induced BMSCs. According to the results of Alcian blue staining, the chondrogenic differentiation (central blue nucleus) of BMSCs was significantly enhanced in the TGF-β1 group, while suppression of p38 could inhibit the chondrogenic differentiation of BMSCs (Fig. 3b).

### Inhibition of p38 signals suppressed the p38/ERK/JNK pathway in the presence of TGF-β1-induced chondrogenesis

**Fig. 4:**
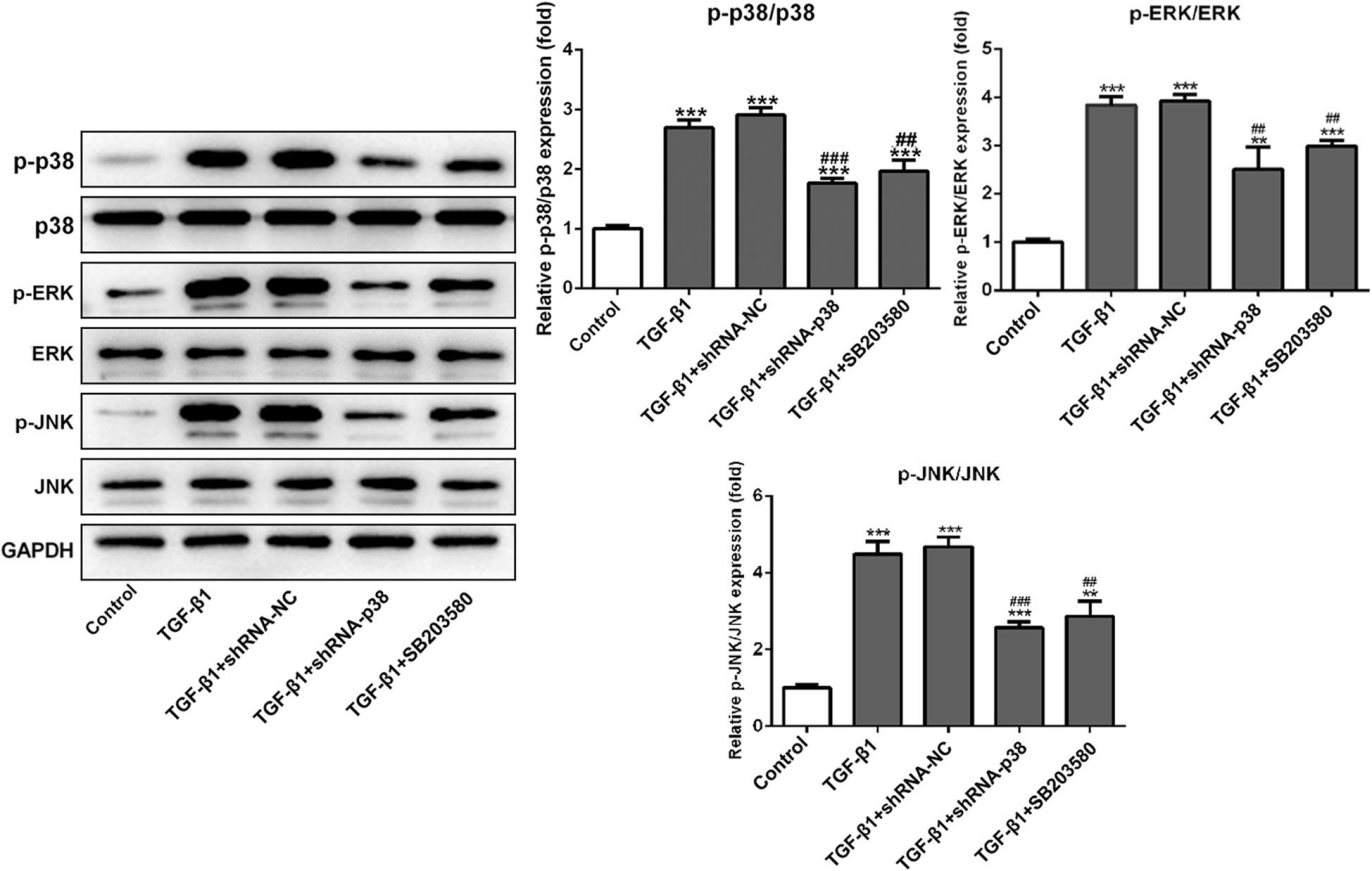
Knockdown of p38 suppressed the activation of p38/ERK/JNK signal pathway in TGF-β1-induced BMSCs. Western blot showed that the expression of p-p38, p-ERK, and p-JNK was remarkably increased in the BMSCs under TGF-β1 induced for 14 days, while shRNA-p38 and SB203580 suppressed the activation of p38/ERK/JNK signal pathway in TGF-β1-induced BMSCs. The non-phosphorylated proteins of p38, ERK, and JNK were essentially unchanged in different groups. **p < 0.01 and ***p < 0.001 vs. control; ^##^*p* < 0.01 and ^###^*p* < 0.001 vs. TGF-β1

The activation of p38/ERK/JNK was assessed using Western blot after treating with p38 inhibitors in TGF-β1-induced BMSCs. The results in Fig. 4 indicated that the expressions of p-p38, p-ERK, and p-JNK were overexpressed compared with the control group (*p* < 0.001). Both shRNA-p38 and SB203580 could decrease the expression of p-p38, p-ERK, and p-JNK in TGF-β1-induced BMSCs. Therefore, these results showed that p38 may promote chondrogenic differentiation by regulating p38/ERK/JNK signal pathways.

## Discussion

Adult human MSCs are considered as promising candidate cell sources for use in cartilage tissue engineering applications. In this study, we further clarify the involvement of intracellular signaling in the regulation of BMSC cartilage formation. The TGF-β1-induced micromass culture system was employed in order to analyze the roles of p38 in the chondrogenic differentiation of BMSCs. This study demonstrated that p38/ERK/JNK pathway was activated by TGF-β1 in chondrogenesis of BMSCs, and played crucial roles in gene transcription of cartilage-specific markers coding for Aggrecan, SOX9, collagen II, Smad1/4, and sGAG. The study also provided evidences that p38 might regulate TGF-β1-induced chondrogenesis and directly interact with p38/ERK/JNK pathway.

In terms of osteogenic differentiation, previous studies indicated that p38 and ERK were involved in bone morphogenetic factor-9-induced osteogenic differentiation of rat dental follicle stem cells [22]. Particularly, p38/MAPK was accepted as a positive regulator in TGF-β-induced BMSCs [23]. In the present study, p38 signal was activated in chondrogenic differentiation of the BMSCs induced by TGF-β after 14 days. The results of Western blot revealed that both shRNA-p38 and SB203580 could decrease the p-p38 expression in TGF-β-induced BMSCs. These results were consistent with previous conclusions. However, the function of the ERK signal remains complex and inconclusive. Prior study reported that cyclic tensile stress may enhance the osteogenic differentiation of periodontal ligament cells via the ERK signaling pathway, and the inhibition of ERK may inhibit osteogenic differentiation [24]. In another study, inhibition of p38 may suppress the osteogenic differentiation of dental pulp stem cells (DPSCs), whereas inhibition of ERK demonstrated the opposite effect [25]. In general, differences between cell type, chondrogenic culture systems, chondrogenic developmental stages, and inhibitor concentrations may be the reason that leads to variations between these results, and diverse methods for detection of chondrocyte differentiation were also regarded as critical factors to explain conflicting results due to differences in accuracy and efficiency [26]. In this study, hBMSCs were derived from BM of healthy adults, and a micromass chondrogenic culture system was employed. The methods have been validated by previous studies in micromass cultures of chick limb bud mesenchyme, pellet cultures of adult MPCs, and mouse chondrogenic ATDC5 cells [27–29]. Prior study revealed that phosphorylation of p38 was relatively stable and prolonged in chondrogenesis stimulated by TGF-β1, but the ERK subtype was phosphorylated in a rapid and transient manner [26]. In our study, the hMSC detection was optimized for a relatively appropriate duration of 14 days. It was found that the expressions of p-ERK, p-p38, and p-JNK were overexpressed in TGF-β1-induced hBMSCs, which could be reversed by p38 inhibitors. This may explain why ERK phosphorylation was relatively difficult to detect.

Chondrogenesis progresses with the expression of a cascade of stage-specific markers. Sox9 protein has been proven to be a master regulatory factor for chondrocyte differentiation [30]. It can transcriptionally activate cartilage-specific marker gene coding for collagen II and regulate synthesis of Aggrecan [31]. In our study, TGF-β1-treated cultures exhibited higher expression in collagen II, Aggrecan, and SOX9 after 14 days compared with the untreated controls, which were decreased by pretreating p38 inhibitors. Moreover, TGF-β1 induced the chondrogenic differentiation of MSCs both in monolayer and in the collagen-GAG scaffold. In previous study, the treatment of MSC-seeded collagen-GAG scaffolds with TGF-β resulted in an increase in [^35^S] sulfate incorporation which was reduced upon inhibition of the p38 pathway [18]. In our study, inhibition of the p38 led to a significant reduction of sGAG synthesis by Alcian blue staining and sGAG assay detection, which were consistent with the previous studies. Therefore, our study indicated that TGF-β1-treated BMSCs differentiated into chondrocytes with expression of chondrocyte-specific genes, and the p38 pathway delicately regulated chondrogenesis induced by TGF-β1.

In previous study, only gene expression of Smad2/3 was examined, and further studies of Smad proteins should be investigated for the interaction between MAPK and Smad signaling on TGF-β stimulation [32]. Interactions between p38 pathways and Smad1/4 were investigated in the process of chondrogenesis stimulated by TGF-β1. TGF-β-induced Smad1/4 overexpression was reduced by inhibition of p38 with shRNA-p38 or SB203580, leading to the downregulation of chondrocyte-specific genes such as collagen II and Aggrecan. Therefore, our findings confirmed that p38 MAPK signal might regulate chondrocyte-specific genes by mediating interaction between TGF-β1 and Smad1/4 molecules. This may provide a new perspective on the control of TGF-β-dependent transcriptional activation in the process of chondrogenesis.

## Conclusion

In conclusion, the present study proved that p38 signals were activated in TGF-β-induced BMSCs, and inhibition of p38 regulated the chondrogenic-specific genes such as SOX9, collagen II, Aggrecan, and sGAG level, through p38/ERK/JNK pathway in TGF-β-induced BMSCs. Meanwhile, it was observed that cross-talk mechanisms between p38 and Smad1/4 pathways were involved in transcriptional regulation of chondrocyte-specific genes stimulated by TGF-β1. These results will deepen our understanding of the mechanism of chondrogenesis process, which provided clues for the application of multipotential stem cells and the treatment of osteoarthritis.

## Data Availability

The datasets used and/or analyzed during the current study are available from the corresponding author on reasonable request.

## Abbreviations

BMSCs: Bone marrow mesenchymal stem cells
ERK: Extracellular signal-regulated protein kinase
GAG: Glycosaminoglycans
JNK: C-Jun N-terminal kinase
OA: Osteoarthritis
TAK1: TGF-β-activated kinase 1
